# Interleukin 18 in the CNS

**DOI:** 10.1186/1742-2094-7-9

**Published:** 2010-01-29

**Authors:** Silvia Alboni, Davide Cervia, Shuei Sugama, Bruno Conti

**Affiliations:** 1 Department of Biomedical Sciences, University of Modena and Reggio Emilia, Italy; 2 Department of Environmental Sciences, University of Tuscia, Viterbo, Italy; 3 Department of Physiology, Nippon Medical School, 1-1-5 Sendagi Bunkyo-ku, Tokyo 113-8602, Japan; 4 Molecular and Integrative Neurosciences Department, The Scripps Research Institute, CA, USA

## Abstract

Interleukin (IL)-18 is a cytokine isolated as an important modulator of immune responses and subsequently shown to be pleiotropic. IL-18 and its receptors are expressed in the central nervous system (CNS) where they participate in neuroinflammatory/neurodegenerative processes but also influence homeostasis and behavior. Work on IL-18 null mice, the localization of the IL-18 receptor complex in neurons and the neuronal expression of decoy isoforms of the receptor subunits are beginning to reveal the complexity and the significance of the IL-18 system in the CNS. This review summarizes current knowledge on the central role of IL-18 in health and disease.

## Introduction

Interleukin (IL)-18 was isolated in 1995 as a co-factor that, in synergism with IL-12, stimulated the production of gamma interferon (INF-γ) in Th1 cells [1]. Since then extensive *in vitro *and *in vivo *studies have identified IL-18 as an important link between innate and adaptive immune responses and a regulator of both cellular and humoral immunity [2-4]. Constitutively produced as an inactive precursor by several cell types IL-18 is secreted in its active form following maturation by caspase 1 in response to inflammatory and infectious stimuli. In addition to its effects on Th1 cells, IL-18 is a strong stimulator of the activity of natural killer cells alone or in combination with IL-15, and of CD8^+ ^lymphocytes. Together with IL-2, IL-18 can also stimulate the production of IL-13 and of other Th2 cytokines. Thus, it is perhaps not surprising that IL-18 was found to be associated with or demonstrated to contribute to numerous inflammatory-associated disorders. These include infections, autoimmune diseases, rheumatoid arthritis, cancer, as well as metabolic syndrome and atherosclerosis [5-11].

IL-18 had not originally been expected to cross an intact blood brain barrier and its immunological effector cells are not normally found in the healthy brain. Yet, studies on the possible role of IL-18 in the central nervous system (CNS), initiated soon after its cloning, were prompted primarily by its similarities with IL-1, which was already demonstrated to have central action. It was soon found that IL-18 could be synthesized centrally and its receptor subunits were now demonstrated to be broadly expressed in neurons. When recombinant interleukin 18 became available it also became clear that IL-18 was active centrally. Work on mice null for IL-18 or its receptor subunit alpha is helping to decipher the action of this cytokine in the brain. Finally, the recent discovery of novel IL-18 receptor subunits in the brain has revealed the complexity of the IL-18 system and may lead to better understanding of both the similarities and opposing actions of IL-1 and IL-18. This review summarizes more than a decade of work aimed at understanding how the IL-18 system contributes to local central inflammatory processes or can influence neuronal function and behavior. A summary of the literature supporting the involvement of IL-18 in neurophysiological and neuropathological conditions is presented in Table 1.

**Table 1:** Representative neurophisiological and neurophatological conditions involving IL-18

Condition	Species	Citation
***Behavior***		

**Sleep**	Rat/Rabbit	[72]
**Fever**	Mouse	[73,162]
**Feeding**	Mouse	[10,11]
**Learning and memory**	Mouse	[77]
	Rat	[48,74,75]
	Human	[108,111,163]

***Stress and HPA axis***		

	Rat	[56,57,81]
	Rat/Mouse	[62]
	Holstein cattle	[80]
	Pig	[54,55]
	Human	[136]

***Neuroinflammation***		

**Brain injury**		
Hypoxia-ischemia	Mouse	[67,84,164-167]
	Rat	[67,168]
Thromboembolic stroke	Mouse	[83]
Spinal cord injury	Rat	[87]
Focal brain ischemia	Rat	[86]
Stroke	Mouse	[59,85]
	Human	[169]
**Nerve injury**	Rat	[47]

**Viral infection**	Chicken	[170]
	Human	[59,171]

***Autoimmune neurodegenerative disease***		

**Multiple Sclerosis**	Human	[95-99,101]
**EAE**	Mouse	[91,93,100]
	Rat	[89,90,92,94]

***Neurodegenerative disease***		

**Alzheimer's disease**	Human	[50,106-109,111-114]
**Parkinson's disease**	Mouse	[117]

***Neuropsychiatric disorders***		

**Depression**	Rat	[133]
	Human	[136,137,139]
**Schizophrenia**	Human	[134,135]

***Other central actions***		

**Excitotoxic damage**		
Ataxia	Mouse	[53]
Neurodegeneration	Mouse	[150]
**Glioma**	Rat	[156,157]
	Mouse	[152-155]

### Components of the IL-18 system

IL-18 is synthesized as an inactive 24-kDa precursor protein that is subsequently processed by caspase-1 into its mature secretable form, which has a molecular weight of 18 kDa [4,12-16]. Pro-IL-18 can also be processed into its active form by various extracellular enzymes including protease 3 (PR-3), serine protease, elastase and cathepsin G [17-19]. Only the mature peptide is reported to be biologically active.

The existence of a putative short isoform of IL-18 resulting from alternative splicing removing 57 bp/19 aa was first described in rat adrenal glands (IL-18α) [20] and subsequently in mouse spleens (IL-18s) [21]. Recombinant IL-18s did not display IL-18-like activity in stimulating INF-γ production when tested alone but appeared to have a modest synergistic action with IL-18. To this date this isoform has not been reported in the CNS.

The IL-18 receptor (IL-18R) belongs to the interleukin 1 receptor/Toll like receptor superfamily. It is comprised of two subunits, IL-18Rα (also known as IL-1Rrp1, IL-18R1 or IL-1R5) and IL-18Rβ (also termed IL-18RacP, IL-18RII or IL-1R7) both with three extracellular immunoglobuling-like domains and one intracellular Toll/IL-1 receptor (TIR) domain [22,23]. IL-18 is believed to bind directly only to IL-18Rα with signal transduction occurring after recruitment of IL-18Rβ to form a high-affinity heterotrimeric complex with IL-18Rα/IL-18 [23-25].

Isoforms of both IL-18Rα and IL-18Rβ were recently described *in vivo *in the CNS. They include a short transcript for IL-18Rα encoding for a receptor subunit lacking the TIR domain arbitrarily named IL-18Rα type II [26]. Since the TIR domain is required for signaling, IL-18Rα type II was proposed to be a decoy receptor, similar to the type II IL-1R [27]. In addition, a truncated form of IL-18Rβ comprising only one of the three immunoglobulin domains was described in rat and human tissues including the brain [28,29]. This form was proposed to act as a soluble negative regulator of IL-18 action by stabilizing IL-18 binding to IL-18Rα yet preventing signaling.

Another negative regulator of IL-18 action is the IL-18 binding protein (IL-18BP). Isolated as cytokine-binding molecules, this 38-kDa soluble protein displays some sequence homology with IL-18Rα [30-32]. IL-18BP binds selectively and with high affinity to mature IL-18, but not to pro-IL-18, preventing its interaction with IL-18Rα. Four human (18BPa-d) and two murine (IL-18BPc and d) IL-18BP isoforms have been described [33]. Of these human IL-18BPb and d lack the structural requirement to inhibit IL-18 action and their role remains to be determined [5].

A different member of the IL-1 family, IL-1F7, is also a negative regulator of IL-18 action. IL-1F7 is able to bind IL-18BP and the IL-18BP/IL-1F7 complex can interact with the IL-18Rβ chain preventing the formation of the funtional IL-18R complex [34]. Several human IL-1F7 splice variants (IL-1F7a-e) have been described [35-39] whereas no murine homologue of IL-1F7 has yet been found. Of these, IL-1F7b (also known as IL-1H, IL-1H4 and IL-1RP1) matured by caspase-1 is capable of binding IL-18Rα [37,40]. Yet, the IL-1F7/IL-18Rα complex failed to recruit IL-8Rβ and no direct agonistic nor antagonistic activity of IL-1F7b for IL-18R was described [37,40].

### IL-18 signaling

**Figure 1:**
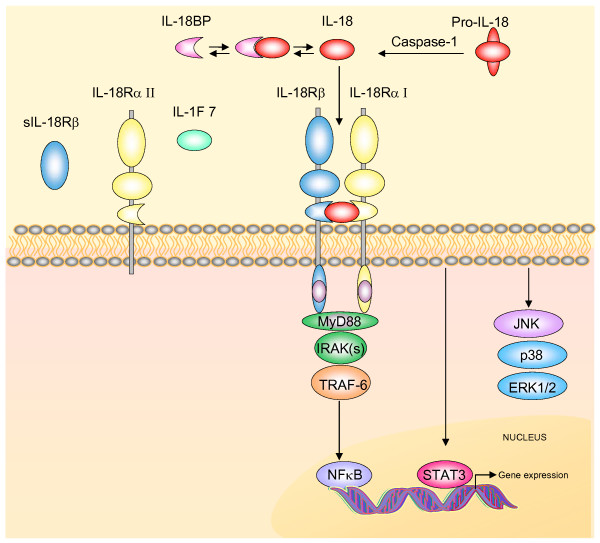
**The IL-18 system**. Active IL-18 is produced and secreted after proteolitic cleavage of the biological inactive precursor Pro-IL-18 by caspase-1. IL-18 action can be regulated by the IL-18 binding protein (IL18-BP) that binds IL-18 with high affinity and inhibits its function. Free IL-18 binds to a specific heterodimeric cell surface receptor, a member of the IL-1 receptor/Toll like receptor superfamily comprised of two subunits, IL-18Rα (here referred to as IL-18RαI) and IL-18Rβ, both with three extracellular Ig-like domains and one intracellular portion containing the Toll/IL-1R domain (TIR). Interaction of IL-18 with the IL-18Rα stabilizes its interaction with IL-18Rβ and with the adaptor protein MyD88 via the TIR domain. This initiates signal transduction by recruitment of the IL-1 receptor activating kinase (IRAK). IRAK autophosphorylates and dissociates from the receptor complex subsequently interacting with the TNFR-associated factor-6 (TRAF6) eventually leading to nuclear translocation of the nuclear factor κB (NF-κB). Engagement of the IL-18R complex can also activate STAT3 and the mitogen-activated protein kinase (MAPK) p38, JNK and ERK. One truncated variant of IL-18Rα (IL-18RαII) lacking the intracellular TIR domain, and one soluble isoform of the IL-18Rβ (sIL-18Rβ) were demonstrated *in vivo *in the mouse brain and in the rat and human brain, respectively. These isoforms originating from differential splicing are proposed to be decoy receptors and possible negative regulators of IL-18 fuction. IL-1F7 is another proposed regulator of IL-18 action (see text for details).

Canonical IL-18 action occurs via recruitment of the adaptor myeloid differentiation factor (MyD88). This event allows activation of the IL-1R-associated kinase (IRAK)/tumor necrosis factor receptor-associated factor 6 (TRAF6) pathway leading to nuclear translocation of the nuclear factor kappa beta (NF-κB) and subsequent modulation of gene transcription [4,5,41,42] (Fig 1).

IL-18 has also been reported to signal via the activation of the transcription factor tyk-2 [43], STAT3 [44] and NFATc4 [45]. In addition, a role for mitogen-activated protein kinases (MAPK) (i.e., extracellular signal-regulated kinase, ERK1/2 and p38), and phosphatidylinositol-3 kinase (Pi3K) in IL-18 signalling has been suggested [4,44-46].

While these data on peripheral cells were consolidated over a decade of work with the immune system, knowledge of the IL-18-dependent signaling in the CNS is only beginning to emerge. Activation of the IL-18R increased NF-κB phosphorylation and induced hypertrophy in astrocytes [47]. In the rat dentate gyrus, the functional effects of IL-18 were significantly attenuated by prior application of c-jun-n-terminal kinase (JNK) pathway, cyclooxygenase-2 (COX-2) and inducible nitric oxide synthase (iNOS) inhibitors, and a role for p38 MAPK was also suggested [48,49]. Moreover, human neuron-like differentiated SH-SY5Y neuroblastomas exhibited an IL-18-dependent increase in the levels of several kinases including p35, Cdk5, GSK-3beta, and Ser15-phosphorylated p53 [50].

### IL-18 system in the CNS

IL-18 transcript was demonstrated by RT-PCR in a variety of brain regions including the hippocampus, the hypothalamus and the cerebral cortex [51,52]. In *in vivo *studies, IL-18 protein was demonstrated in the pituitary gland, ependymal cells, the neurons of the medial habenula (where its synthesis was elevated by stress), in Purkinje cells, and in astrocytes in the cerebellum [53-57]. In addition, it was demonstrated *in vitro *that microglia and astrocytes can produce IL-18 [58-61] and its level can be up-regulated following LPS stimulation [62] or treatment with INF-γ [63].

In the CNS, Northern blot analysis failed to detect the presence of IL-Rα [64] and IL-Rβ [65] soon after their cloning. The first evidence that IL-18R components are expressed in brain tissue was obtained by Wheeler and colleagues [52] which reported the constitutive expression of IL-18Rα, IL-18Rβ in the rat hypothalamus by RT-PCR. Subsequently, the mRNA expression of IL-18Rα, IL-18Rβ and the soluble form of the IL-18Rβ were detected in the hypothalamus, hippocampus, striatum and cortex and in cultured astrocytes, microglia and neurons [28]. More recently *in vivo *analysis showed that IL-18Rα mRNA and protein are constitutively expresseed in neurons throughout the brain [26,53,60,66]. Similar neuronal localization and distribution was found for IL-18Rβ (Alboni et al., unpublished data). At the same time it was demonstrated that the truncated decoy form of the IL-18Rα was expressed in neuronal cells with a pattern similar to that of its active counterpart [26]. Overall, IL-18R subunits had broad distribution across the brain with the highest level in the hypothalamus, hippocampus and amygdala. Finally, both IL-18 and IL-18R subunits are inducible and their CNS levels can be regulated. For instance, in the mouse hippocampus the levels of IL-18 and IL-18Rα increased after kainic acid (KA)-induced excitotoxicity, [60] whereas hypoxic-ischemic brain injury markedly increased IL-18 expression in mouse microglia [67]. In addition nerve injury induced IL-18 upregulation in rat spinal cord microglia possibly via p38 activation [47].

IL-18BP has been investigated and demonstrated in rodent brains, mixed glia and microglia by only one group using RT-PCR. Its distribution and action in the CNS remain to be investigated [52,68].

Information on central IL-1F7 is also limited to one study that demonstrated its presence in the human brain [37]. Investigating IL-1F7 in the CNS is also hampered by the fact that a mouse homologue has not been identified.

### Behavior

The similarities between the IL-1 and the IL-18 systems suggested the possibility that like IL-1β, IL-18 may be one mediator of the behavior symptoms of sickness. These include fever, lethargy, hypophagia and cognitive alterations [69-71].

It was demonstrated that central intracerebroventricular (i.c.v.) injection of IL-18 in rabbits and rats increased non-rapid eye movement sleep as well as brain temperature [72]. The lethargic effects of IL-18 were also observed following intraperitoneal injection of IL-18, whereas, unlike IL-1β, peripheral administration did not induce fever [72,73]. Instead, pre-treatment with IL-18 reduced the pyrogenic effects of IL-1 suggesting the possibility of an antagonizing effect of these cytokines on fever [73].

Work on IL-18 null, and on IL-18BP overexpressing mice indicated that IL-18 is anorexigenic and can modulate feeding, but also energy homeostasis, influencing obesity and insulin resistance [10,11]. The mechanisms through which IL-18 exerts these effects are largely unknown but central action was proposed following the observation that i.c.v. injections of exogenous IL-18 induces sleep [72] and anorexia [11]. The recent demonstration that IL-18 functional and regulatory subunits of the IL-18R are expressed in several brain regions including the hippocampus, the hypothalamus and the cortex provided a molecular and cellular basis for the central action of IL-18 in modulating these functions [26].

Evidence for the role of IL-18 as modulator of neuronal functions includes studies on the hippocampal system, a structure that plays a major role in memory and in cognition. For instance, IL-18 reduces long term potentiation (LTP) in the rat dentate gyrus, possibly through the involvement of metabotropic glutamate receptors [48,49,74,75]. In particular, IL-18 had no effect on baseline synaptic transmission or paired pulse depression, but significantly depressed the amplitude of NMDA receptor-mediated field excitatory post synaptic potentials [75] providing evidence of a direct neuromodulatory role for IL-18 in synaptic plasticity. It is possible that IL-18 may act directly on the neurons of the dentate gyrus, moreover, its action may be regulated by the relative level of IL-18Rα type I and type II, both highly expressed in these cells [26]. Work on CA1 pyramidal neurons of mouse hippocampal slices demonstrated that IL-18 stimulated synaptically released glutamate and enhanced postsynaptic AMPA receptor responses, thereby facilitating basal hippocampal synaptic transmission without affecting LTP [76].

Recently an *ex vivo *study found that LPS-induced IL-18 elevation in the brain was unable to affect LTP in the CA1 hippocampal subregion [77]. However, when comparing wild-type and IL-18 KO mice, the same study demonstrated that IL-18 regulates fear memory and spatial learning. In particular, assessment of spatial learning and memory with the water maze test showed that compared to wild-type mice IL-18 KO mice exhibit prolonged acquisition latency and that this phenotype was rescued by i.c.v. injection of IL-18.

### Stress and the hypothalamic-pituitary-adrenal axis

IL-18 occupies a peculiar role in stress response both centrally and peripherally. This subject was recently extensively reviewed and we briefly refer to it here only for those aspects relevant to understanding of the central actions of this cytokine [78].

In response to restraint stress, IL-18 null mice showed a markedly reduced morphological microglial activation in the thalamus, hypothalamus, hippocampus, substantia nigra and central gray area [62]. In addition, IL-18 expression was elevated by restraint stress in the neurons of the medial habenula [56]. Since the habenula is a potential site for the interaction of neuro-endocrine and immune functions, the possibility exists that IL-18 might mediate the communication between the CNS and the periphery. Indeed, there is a general agreement that IL-18 may regulate hypothalamic-pituitary axis activity, possibly mediating the stress response of the adrenal gland [20,78,79]. In this respect, stress has been shown to induce transient IL-18 mRNA elevation in rat pituitary cells where increase of the IL-18 mRNA level was observed also after adrenalectomy [57].

IL-18 is also produced in the neurohypophysis [20] as well as in the adenohypophysis where *in situ *hybridization combined with immunohistochemistry demonstrated its expression in corticotrope cells [57]. In addition, bovine somatotropes have been shown to produce IL-18 and IL-18Rα was co-localized with IL-18, or growth hormone, suggesting the possibility that IL-18 acts on somatotropes through the autocrine pathway [80]. However, IL-18 seems to also act at the hypothalamic level. Indeed, the application of IL-18 in rat hypothalamic explants decreases basal and KCl-stimulated corticotropin-releasing hormones (CRH), as well as CRH gene expression [81]. In particular, the cytokine did not modify basal PGE2 production but abolished production stimulated by IL-1β demonstrating that IL-18 possesses a profile of *in vitro *neuroendocrine activities opposed to, and even antagonizing, those of IL-1β. Recently, IL-18 was localized in the marginal cell layer of the bovine and porcine Rathke's pouch, that is assumed to embody a stem/progenitor cell compartment of the postnatal pituitary gland [54,55]. Interestingly, stimulation of a cloned anterior pituitary-derived cell line (from the bovine anterior pituitary gland) with IL-18 increased expression of mRNAs of a different cytokine suggesting the possibility that IL-18 may modulate not only the immuno-endocrine function of the pituitary cells but also their development [55].

### Microglia and neuroinflammation

Functional maturation and activation of IL-18 can occur in the brain under inflammatory conditions. Indeed, as extensively reviewed by Felderhoff-Mueser and colleagues [82], experimental and clinical studies suggest that binding of IL-18 occurs in several neuroinflammatory associated pathological conditions including microbial infections, focal cerebral ischemia, Wallerian degeneration and hypoxic-ischemic, hyperoxic and traumatic brain injuries (e.g., stroke). Further evidence comes from recent papers reporting an activation of IL-18 in the brain of mice that underwent thromboembolic stroke [83] or an increase of IL-18 levels after hypoxia-ischemia in the juvenile hippocampus of mice [84].

During CNS inflammation, the IL-18 system may have an important role in the activation and response of microglia and possibly infiltrating cells. As mentioned above, microglia cells can synthesize and respond to IL-18 [20,59-61]. IL-18 KO mice had impaired microglia activation with reduced expression of Ca^2+-^binding protein regulating phagocytic functions that resulted in reduced clearance of neurovirulent influenza A virus [85]. In the absence of infection IL-18 deficient mice also showed diminished stress-induced morphological microglial hypertrophy [62].

Interestingly, IL-1β is upregulated within 4 h of focal ischemia in rat brain, but IL-18 is upregulated much later, at time points associated with infiltration of peripheral immune cells, thus suggesting different roles for these interleukins in the regulation of glial functions [86]. In this respect, it was shown that mice infected with Japanese Encephalitis produce IL-18 and IL-1β from microglia and astroglia [59]. Both interleukins are capable of inducing pro-inflammatory cytokines and chemokines from human microglia and astroglia, although IL-18 seems to be more potent than IL-1β.

In the spinal cord, IL-18 seems to play a role in the innate inflammatory response. Indeed, moderate cervical contusive spinal cord injury induced processing of IL-18 in neurons of the rat spinal cord [87]. In addition, nerve injury induced a striking increase in IL-18 and IL-18R expression in the dorsal horn, and IL-18 and IL-18R were upregulated in hyperactive microglia and astrocytes, respectively [47,88]. Intrathecal injection of IL-18 induced behavioral, morphological, and biochemical changes similar to those observed after nerve injury [47], suggesting that IL-18-mediated microglia/astrocyte interactions in the spinal cord have a substantial role in the generation of tactile allodynia.

### Autoimmune neurodegenerative disease

A pivotal role for IL-18, in the pathogenesis of autoimmune neurodegenerative disease has been proposed. High levels of IL-18 mRNA were found in the brain and the spinal cord of rats with experimental autoimmune encephalomyelitis (EAE), an animal model of multiple sclerosis (MS) [89,90]. Elevated IL-18 transcript was found at the onset and throughout the course of the disease. A different study showed that IL-18 increases severity of EAE [91]. Moreover, it has been demonstrated that anti-IL-18 antibodies or targeted overexpression of IL-18BP in the CNS had preventive effects on the induction of EAE[90,92]. These observations suggested a role for IL-18 in MS, IL-18 KO mice were susceptible to EAE, whereas IL-18Rα KO mice or IL-18 KO mice treated with anti-IL-18Rα antibodies were not [93]. Thus, alternative IL-18Rα ligands with encephalitogenic properties may exist [93]. In EAE-susceptible Dark Agouti rats, the basal and post-immunization (day 5, 7 and 12) levels of IL-18Rα in lymph node cells were significantly higher than in the EAE-resistant Piebald Virol Glaxo rats [94].

In human, serum and cerebrospinal fluid levels of IL-18 are elevated in patients with MS [95-98] and IL-18 positive cells have been detected in demyelinating brain lesions from MS patients [99].

The pathological role of IL-18 in EAE is also supported by the up-regulation of caspase-1 (required to convert IL-18 precursor protein into its biologically active mature form) mRNA in the spinal cord of rats with EAE [89], and decreased disease severity in caspase-1 KO mice [100]. Finally, peripheral blood mononuclear cells from patients with MS have elevated caspase-1 mRNA levels [95,101].

In addition to a role in MS there is also evidence to support a function for IL-18 in the onset and progression of autoimmune CNS disease. For instance, infection of microglia lines with Theiler's murine encephalomyelitis virus (which causes the development of a chronic-progressive autoimmune demyelinating disease) significantly upregulates the expression of cytokines involved in innate immunity, including IL-18 [102].

### Neurodegenerative disorders

Alzheimer's Disease (AD) is the most common type of human dementia. It is characterized clinically by a gradual but progressive decline in memory and pathologically by neuritic plaques, neuro-fibrillary tangles, and the loss of synapses and neurons [103]. Inflammatory processes were proposed to contribute to neurodegeneration in AD and extensive studies indicated that IL-1 is a pivotal cytokine in mediating direct neuronal loss and sustaining microglia activation leading to further cellular damage in AD [104]. Microglia-derived inflammatory cytokines can initiate nerve cell degeneration and enhance the plaque production typically found in AD [105]. Increasing evidence indicates that IL-18 may have a role in this scenario.

For instance, the levels of IL-18 transcript and protein were increased in the frontal lobe of AD patients compared to healthy age-matched controls. In these brains IL-18 was found in microglia, astrocytes and in neurons that co-localize with amyloid-β-plaques and with tau [106], suggesting that amyloid-β may induce the synthesis of IL-18, and IL-18 kinases involved in tau phosphorylation as a part of the amyloid-associated inflammatory reaction. Additionally, IL-18 can enhance protein levels of Cdk5/p35 and GSK-3β kinases, tau phosphorylation and cell cycle activators in neuron-like differentiated human SH-SY5Y neuroblastoma cells [50]. Thus, on a pathway leading to AD, IL-18 may have an impact on the hyperphosphorylation of tau but also on cell cycle related mechanisms. In the plasma, the levels of IL-18 were significantly elevated in patients with AD, vascular dementia, and mild cognitive impairment compared to the control group [107,108]. Interestingly, IL-18 levels were higher in AD-mild patients, were slightly lower in AD-moderate patients, whereas no significant difference was observed between AD-severe patients and non-demented age-matched subjects [109], suggesting a gradual decline of immune responsiveness in AD. Although other studies showed no differences in circulating IL-18 levels measured between AD patients (both mild cognitive impairment and severe AD patients) and controls [106,110,111], a significant increased production of IL-18 was obtained from stimulated blood mononuclear cells or macrophages of peripheral blood of AD patients [111,112]. Furthermore, a significant correlation between IL-18 peripheral production and cognitive decline was observed in AD patients. Overall, these data indicate that IL-18-related inflammatory pathways, are exacerbated in the peripheral blood of AD patients, and that this cytokine may indeed participate in pathogenic processes leading to dementia.

Genetic association studies reported that two functional polymorphisms (137G/C and -607C/A) in IL-18 promoter may increase the risk of developing sporadic late onset AD in the Han Chinese population [113]. An association between 137G/C and -607C/A polymorphisms and the susceptibility/clinical outcome of AD was also suggested in an Italian population [114], although these correlations remain controversial. Indeed, in another Italian population a lack of association between IL-18 gene promoter polymorphisms and onset of AD was reported, indicating that the association of IL-18 promoter polymorphisms with AD is not so strong, AD being a multifactorial disease [115]. Importantly, IL-18 promoter remains poorly characterized.

Finally, it has been hypothesized that increased production of IL-18 in the brain may lead to motor and cognitive dysfunctions, leading to the development of HIV-associated dementia. Thus, IL-18 concentrations in HIV-infected persons are likely to play an important role in the development and progression of the infection toward AIDS and associated clinical conditions [116].

In Parkinsonism, there is evidence of chronic inflammation in the substantia nigra and striatum. Activated microglia, producing proinflammatory cytokines, surround the degenerating dopaminergic neurons and may contribute to dopaminergic neuron loss. In an experimental model of Parkinson's disease that utilized injection of the dopaminergic specific neurotoxin MPTP the number of activated microglial cells in the substantia nigra pars compacta of IL-18 KO mice was reduced compared to wild-type [117], indicating the possibility that IL-18 may participate in microglial activation and dopaminergic neurodegeneration.

### Neuropsychiatric disorders

Several groups found that depressed and schizophrenic patients have high circulating levels of pro-inflammatory cytokines [118-123]. Others reported that psychotic episodes often occur in conditions characterized by elevated levels of pro-inflammatory cytokines, for instance during inflammation or in patients suffering from immune diseases [124-127].

Other studies suggested that the correlation between inflammatory markers and psychiatric disorders may be more that merely associative, with inflammation actually contributing to mental disorders. Improvement in psychiatric symptoms has been recently reported in patients treated with anti-inflammatory drugs for other indications [128] and functional allelic variants of genes codifying for pro-inflammatory cytokines were associated with reduced responsiveness to antidepressant therapy [129,130]. It was also recently demonstrated that IL-6 plays a pivotal role in the pharmacological ketamine model of schizophrenia by modulating the NADPH-oxidase increase of superoxide affecting parvalbumin interneurons [131]. An interesting line of research is exploring the possibility that these actions may be developmental, with cytokines influencing early-life programming of brain functions [132].

At present evidence linking IL-18 and psychiatric disorders are primarily associative. IL-18 mRNA expression is elevated in subordinate rat models with depression with respect to dominant rats [133]. A significant elevation of circulating plasma levels of IL-18 has been reported in subjects affected by schizophrenia and were normalized by pharmacological treatment with risperidone, a dopamine antagonist with antipsychotic activity [134,135]. Normalization was demonstrated also within 6 month of treatment with the antipsychotic clozepine [134] although the possibility that these effects could be due to clozepine's effects on leukocyte numbers cannot be excluded. The serum levels of IL-18 were also significantly higher in moderate-severe depression patients, further suggesting that the pathophysiology of depression is associated with an inflammatory response involving IL-18 [136,137]. Coincidentally, IL-18 is also elevated after stroke, a condition followed by emotional disorders [138-140].

The significance of these correlations with respect to the role of the IL-18 system to neurophsychiatric disease pathophysiology or manifestation remains to be determined. Caution should be taken particularly since peripheral IL-18 can be subject to neurogenic stimulation or stress [20,78,141-143]. It is thus difficult to determine whether IL-18 elevation contributes to these pathologies or whether it is a consequence of the disorders. Indeed, Kokai and colleagues suggest that IL-18 can be considered a psychologic stress-associated marker since they demonstrated that exposure to stressful events (i.e., panic attack in human, restraint stress in mice), the most important precipitating factor in depression, induces a prompt increase in the level of circulating IL-18 [136].

Regardless, elevated IL-18 levels have the potential to contribute to several of the symptoms associated with neuropsychiatric disorders. For instance, like other pro-inflammatory cytokines, IL-18 may participate in the control of the activity of the HPA axis reported to be dysregulated in depression [78,144-146]. IL-18 may antagonize glucocorticoid signalling via activation of NF-κB and p38 MAPK possibly disrupting glucocorticoid-dependent negative feedback on the HPA axis [147-149]. Finally, IL-18 can affect other hallmarks of depression impairing learning and memory by acting as an attenuator of long-term potentiation, and inducing lethargy and loss of appetite [11,72,74].

### Other central actions of IL-18

Three groups investigated the action of IL-18 in rodents following administration of KA, an agonist of the kainate receptors inducing seizure, cerebellar ataxia and exitotoxic mediated neuronal loss [53,60,150]. In mouse hippocampus KA elevated IL-18 and IL-18R expression on microglial cells progressively 3 days after treatment [60]. The authors hypothesized that similar to what was observed peripherally in studies of the immune system, IL-18 may contribute to cellular damage. This hypothesis was partially supported by another group showing that the KA-induced hippocampal neurodegeneration was shown to be more severe in IL-18 KO mice compared to wild-type littermates [150]. Yet, in recombinant mice with the same pre-treatment, IL-18 aggravated both the clinical and pathological signs of neurodegeneration in a dose-dependent manner.

In the cerebellum, where KA was demonstrate to induce ataxia partially via elevation of IL-1β, exogenous IL-18 was protective and played a positive role in the recovery from kainate-induced ataxia [53]. Consistently, IL-18 KO and IL-18Rα KO mice show delay in recovery from kainate-induced ataxia. The antagonizing effects of IL-18 and IL-1β also observed in the peripheral effects of IL-1β on fever, [73] are intriguing particularly since these cytokines share many similarities including their signalling. Preliminary observations suggest that these effects may be explained by IL-1β and IL-18 targeting different cells or activating distinct signalling [53].

Some groups have investigated the possibility that IL-18 could be used against glioma, a common and highly aggressive type of brain tumor with poor long-term prognosis [151]. In this respect, IL-18 was investigated alone or in synergism with IL-12 or Fas, for its ability to induce INFγ and NO inducing a cytotoxic response against glioma cells [152]. Systemic or intracerebral administration of IL-18 inhibited the growth of inoculated glioma cells and prolonged the survival of mice with subcutaneous or brain tumors, respectively [153]. Antitumor activity against glioma was also found in mice treated with IL-18 and IL-12 via Semliki Forest virus [154,155] or with a combination of IL-18 and Fas [156]. Finally, encouraging data were also reported by overexpressing IL-18 in mesenchymal cells of rats [157].

## Conclusions

Investigation on the presence of IL-18 in the CNS began soon after its discovery as a co-stimulator of INF-γ production in the immune system [1,20,158,159]. Initially IL-18 was investigated for its similarities with IL-1β as a possible mediator of sickness behavior and of local inflammatory reactions associated with neuronal damage. These actions were both demonstrated and IL-18 was shown to promote loss of appetite, sleep and inhibition of LTP, as well as to be produced by and active in microglial cells, and to possibly contribute to neurodegenerative diseases.

Yet, two observations suggest that IL-18 has a central role and function that may be unique and distinct from those of IL-1β or other cytokines. The first being the recognition that Il-18 and Il-1β, when their combined action was tested, may have antagonizing effects such as those occurring in fever and in kainate-induced cerebellar ataxia. The second was the finding that IL-18R is constitutively and broadly expressed in neuronal cells throughout the rodent brain. This finding opened the possibility of a direct action of IL-18 on neuronal functions particularly in all of the CNS disorders showed to be correlated to elevated cytokine levels.

Thus, the investigation of the central action of IL-18 may be considered in its infancy and the significance of the neuronal IL-18R complex and of its isoforms remains to be determined. Among the intriguing peculiarities of the central role of IL-18 is that despite the constitutive expression of the receptor, the regulation of IL-18 action appears to be regulated by the existence of truncated isoforms and by the fact that under normal physiological conditions IL-18 is not easily found in the CNS. Interestingly, the genes encoding for IL-18 and its receptors are subject to differential promoter usage and their transcription to differential splicing indicating that these molecules have the potential of being produced in a tissue/cell specific way in response to different stimuli [26,78]. It will be important to determine which physiological or pathological conditions modulate these molecules.

Also unexplored is the investigation of the possible role of IL-18 in CNS development suggested by work on microglial cultures from newborn mice and brain homogenates where IL-18 was preferentially expressed during early postnatal stages and subsequently downregulated, being virtually absent in the brains of adult mice [61]. Additionally, the activated microglia-derived cytokines, including IL-18, may either inhibit the neuronal differentiation or induce neuronal cell death in the rat neural progenitor cell culture, which are cells capable of giving rise to various neuronal and glial cell populations in the developing and adult CNS [160]. Finally, in adult rodents IL-18 is produced in ependymal cells [56] considered a primary source of neural stem cells in response to injury [161]. The possible role of IL-18 in their differentiation has also not been investigated.

Work on existing IL-18 and IL-18R null mice as well as the development of new experimental models including CNS specific null or overexpressor mice and the identification of suitable *in vitro *systems will determine the specificity of the central effects of IL-18 in health and disease.

## Competing interests

The authors declare that they have no competing interests.

## Authors' contributions

SA and DC wrote the initial draft of the manuscript, SS and BC contributed to its final version. All authors read and approved the final manuscript.
